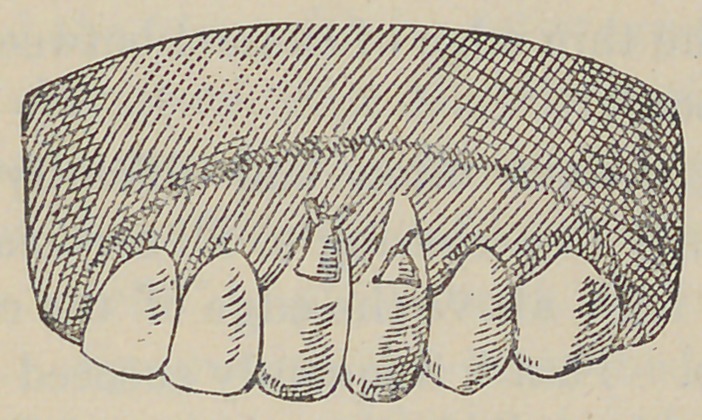# A Means of Holding the Rubber Dam While Operating upon Cavities in Labial and Buccal Surfaces

**Published:** 1891-01

**Authors:** C. R. Butler


### A Means of Holding the Rubber Dam while Operating upon Cavities in Labial and Buccal Surfaces.

BY C. R. BUTLER.

Read before the Ohio State Dental Society, October 29, 1890.

A great number and variety of clamps and other devices have been tried and used by operators, but in many cases to the very great discomfort of the patient. And when there is a high degree of sensitiveness of dentine there is oftentimes very great pain, and sometimes almost beyond endurance. Even if the clamp remains steady after being placed in position, it is not always possible to carry the rubber dam over it. Most operators are able to reach ordinary cases, but the question is how to reach the desperate ones that are occasionally presented. It is to this last variety of cases that I propose to call attention for a short time, and if a method can be suggested to overcome this difficulty, then a long step has been made in securing labial surface cavities against moisture while being filled.

The illustration here presented is from a case in practice which came under my hands about a month ago. Several fillings of cavities of this kind had been made by other operators, and more was needed. The patient expressed much dread of the clamp, but when told that the clamp would not be used said, " well, I can stand any thing else." In this case I put in two small screws, as shown in the cut. One is quite sufficient in most cases.

If the drill is nicely gaged to the screw wire the screw will become firmly attached even in a shallow. Set the screw as near the border of the cavity as may be, and this should be done before the excavation of the cavity. The screw should be set sufficiently distant from the border to avoid the liability of checking the dentine. After the filling is introduced the protruding portion of the screw is cut off and finished upon the surface as a small filling would be. I prefer the hand drill. Never use a screw tap. Let the thread upon the wire be sufficient for cutting its way into the dentine.

The iridio-platinum or gold wire is hard enough to cut its way into the tooth bone, and the smallest size wire made is the best for the purpose. The small drills made by the S. S. White Dental Manufacturing Co., with limit shoulder, are the safest for the operator if he is not able to make them himself. After having introduced the screws, place the dam on the tooth, slipping its edge over these screws before beginning to excavate. It is well to dip the end of the screw in phosphate cream with a trace of creosote added when ready to put it in place.

The illustration accompanying this will illustrate the principle and mode of procedure without further description.

DISCUSSION OF DR. BUTLER'S PAPER.

Dr. J. Taft: The clamps of various patterns devised for application to cervical cavities are rather unsatisfactory, in the main. I have been agreeably surprised to discover how admirably the set of clamps devised by Dr. Delos Palmer fulfill their purpose. They are easily applied and retain their grip upon the teeth without discomfort to the patient and without causing the operator apprehension of their slipping off. I have discarded all the ordinary forms of clamps in favor of this set. My only regret is that I did not long ago examine and discover their excellence. The set is rather expensive [$25.00 (?) Rep.] but I would not be willing to dispense with them for twice or thrice their cost.

Dr. Jennings: The set contains the only wisdom tooth clamp; good for any thing.

Dr. Taft: I have been really surprised to find how well they fit teeth differing in size in different mouths. This method of using screws devised by Dr. Butler, is perhaps good in many cases, but particular care would need to be used to avoid breaking or checking the thin edge of enamel between the screw itself and the cavity's border.

Dr. Fletcher: I have often had difficulty in adjusting the rubber dam properly in preparing to fill cervical cavities. If the dam be forced up high above the edge of the cavity it will often leak at the sides. That I frequently succeed in controlling by wiping into the cavity a little dry plaster of Paris, using a piece of spunk to push it under the edges of the dam where leakage occurs. The moisture combining with the dry powder makes in a minute or two a further barrier to the inflowing secretions.

Dr. H. A. Smith: I think we sometimes become so prepossessed in favor of the dam that we forget all about what is often perfectly practicable in such cases—that is, to dispense with the dam, depending upon simpler means. These cavities frequently can be kept dry long enough with a napkin, or by other means to enable us to fill them perfectly. I often use a holder in conjunction with the dam, or have my assistant use it to carry the dam back and hold the gum away.

Dr. Callahan: I have very little faith in a cohesive gold filling made without the dam. I hold that even the slight danger of moisture from the breath is enough to imperil the cohesive properties of the foil. (Applause). For extreme sensitiveness of these labial cavities I moisten the cavity with an aqueous solution of nitrate of silver; let it go a week or two, when it can be excavated with but little pain. I have been using for some time Dr. W. S. How's cervical border clamp, which accomplishes its purpose admirably.

Dr. Sage: I have experimented to a limited extent with a device which I will try to describe. I draw the temper of an ordinary steel pen, or a three nought [000] file handle will do, then I cut from this a strip a line or two wide, and half or three-fourths of an inch long. I bend this into the shape of a letter U, having first sharpened carefully the middle third of the strip, along one edge. Then restore the temper and polish the inner side so that it will reflect the light. Now take a cavity, labial, in an upper incisor. Having adjusted the dam over it and adjoining teeth, I use the upper curved end of the U to push the dam back (and the gum with it) fully exposing the cavity to view. Press the sharpened edge slightly against the cementum of the root, press over the edges of the teeth a good sized piece of impression compound sufficiently softened for the purpose, and while holding the "crib" in place, work the compound around it, so as to hold it. By pressing the sharpened edge of the crib slightly against the cementum with the thumb of the left hand, while the fingers are holding the impression compound in place, you can burr out the cavity thoroughly and at your leisure, and with no risk of tearing the dam or of wounding the gum. The crib itself excludes seeping moisture while the dam prevents a general flooding.

Dr. Jennings: Many of these labial cavity fillings fail because they are not properly finished. Use stones, disks, burnishers, and so on with the right-angle instrument so as to reach all parts of the filling and finish very thoroughly. For retaining the everted edges of the dam in place use powdered rosin on the tooth.

Dr. Miles: To accomplish that purpose I use a heated punch for making the holes in the rubber. This leaves a gummy edge to the hole so that it retains its position when once turned back.

Dr. Jennings: The objection to the hot punch is that it makes the rubber rotten, so that you can not tell at what moment your dam is going to tear.

[Subject Passed].

Camphor a Solvent for Iodoform.—Camphor increases the solubility of iodoform in alcohol and ether. While one hundred parts of alcohol ordinarily dissolve not more than one and one-fourth parts of iodoform, the same amount of a saturated solution of camphor is capable of taking up as much as ten parts.

## Figures and Tables

**Figure f1:**